# Effect of Generational Status on Child Well-Being: Mediating Effects of Social Support and Residential Instability

**DOI:** 10.3390/ijerph16030435

**Published:** 2019-02-02

**Authors:** Ko Ling Chan, Ruby Lo

**Affiliations:** Department of Applied Social Sciences, The Hong Kong Polytechnic University, Hong Kong, China; rubylo@spr.com.hk

**Keywords:** child health, migration, generation, quality of life, social support

## Abstract

Children in migrant families often encounter difficulties that have great impacts on their health. However, there is a lack of research to examine generational status and child health-related quality of life (HRQoL). This study compared the HRQoL of children, aged 3 to 19 years, born in Hong Kong to mainland parents with second- and third-or-higher-generation children; and explores the mediating effects of residential instability and of social support on the association between generational status and HRQoL. A sample comprised 4807 reports on children (mean age = 7.47 years) in Hong Kong was analyzed. Significantly lower HRQoL related to physical functioning was observed among children in migrant families. Association between generational status and child HRQoL was mediated by commute time between home and school, frequency of moving home, and social support. Findings lend utility to addressing similar issues amongst other developmental immigrant populations.

## 1. Introduction

Migration and the experiences of children in immigrant families often have vast implications for child health. Compared to adults, immigrant children may encounter unique difficulties while adapting to changes during migration, such as acculturation, language barriers, and social exclusion [1]. These stressors have been consistently demonstrated to have deleterious effects on children’s well-being, including worse physical health [2], mental and emotional health [3], academic performance [4], and less access to healthcare services [5]. However, some findings show that immigrants can have better health outcomes across certain domains, such as risk of mortality, injury [6], and psychiatric disorders [7], otherwise coined as “the healthy immigrant effect” [8]. Hence, additional research to elucidate the pathways of risk and resilience in the development of immigrant children are still warranted.

Immigrant children have been typically categorized into different generations: the first generation refers to the immigrants whose parents are foreign-born; the second generation the local-born children of immigrant parents; and the third-or-higher generation the local-born children of two local parents. In general, later generations of children have better physical and psychological well-being [9], as well as higher educational attainment and greater employment rate than the first generation [10]. In the United States, the number of children born to undocumented immigrant parents has increased sharply in recent decades. These children, sometimes referred to as “anchor babies” [11], are born as U.S. citizens, but suffer from worse healthcare outcomes, such as difficulties in obtaining medical insurance coverage and less healthcare utilization in general [12,13]. This growing population of children is currently estimated to be over 5.5 million [14], and the long-arm effects of risk factors unique to their developmental context, as well as possible intervention targets, should continue to be examined.

This study aimed to investigate the link between generational status and children’s well-being by exploring the possible pathways of risk and resilience to health-related quality of life (HRQoL). This study mainly explored the problem of “anchor babies” in Hong Kong. Since 2003, the Hong Kong Government has adopted an Individual Visit Scheme, which targets to boost the economy. Yet, it also provides an opportunity for pregnant women from Mainland China to deliver their babies in Hong Kong either legally through hospital reservation or illegally by showing up at the emergency departments when they are in labor. A social issue arises with the increase in the number of “birth tourists” in Hong Kong: The babies are born with the identity of permanent residents in Hong Kong, while their parents are not. These “anchor babies” could be regarded as migrant children. However, since the children born in Hong Kong to mainland couples are not involved in the migration process, nor are they born to immigrants residing in Hong Kong, they are not official categorized as the first or the second generation, so the classification of “1.75 generation” was adapted from previous research, referring to immigrants who arrived in early childhood (as early as age 0) [10]. Using the such definition, children in Hong Kong could be classified into three groups according to their migration status: (a) the 1.75 generation, in which both parents were not Hong Kong citizens when the child was born (anchor babies); (b) the second generation, in which one parent was a Hong Kong citizen (commonly the father) while the other was not when the child was born; and (c) the third-or-higher generation, in which both parents were Hong Kong citizens. This allowed us to compare risk factors and resilience indicators across three distinct immigrant statuses.

One domain of risk factors is residential instability. Children in the 1.75 or the second generation may not consistently reside in Hong Kong compared to the third-or-higher generation due to the citizen status of their parents. These children may be more likely to reside in areas far from their schools to accommodate cross-boundary home arrangements. For example, some 1.75- or second-generation children need to travel between Hong Kong and mainland China every day [15]. This long commute to school and frequent mobility may lower HRQoL by increasing fatigue levels, limiting time to engage in activities with peers, and reducing opportunities to develop social networks [16].

On the other hand, social support is one resilience dimension that can dampen the effects of immigration and acculturation stress on well-being [17]. While social support has long been demonstrated to promote HRQoL [18,19], some empirical observations have indicated that immigrants may be deprived of social support [20]. Launching from these findings corresponding to risk and resilience mechanisms by which immigration contexts and generation status can potentially shape well-being outcomes and HRQoL, we constructed a model to integrate risk and resilience dimensions in one study and explored (a) the effects of generational status on child HRQoL in three distinct categories of immigrant children, and (b) the mediating roles of residential instability and social support. Based on the findings in the literature [9,12,13], it was hypothesized that generational status would be predictive of child HRQoL, in particular the higher generation would show better HRQoL than lower generations, and this association would be mediated by residential instability and social support via different pathways.

## 2. Materials and Methods

### 2.1. Study Design, Sample, and Procedure

A cross-sectional, school-based survey study was conducted in 2015. This study adopted a two-stage stratified sampling procedure, with stratification at both the level of geographical district and the type of schools in Hong Kong in order to maximize the representativeness of the sample. During the first stage, 107 schools, including kindergartens, primary schools, and secondary schools among 18 districts in Hong Kong were sampled. In the second stage, eligible children, who aged 3 to 19 years, from the schools were randomly sampled and asked whether they agreed to participate. In this study, all children who were born in Hong Kong and receiving education in the sampled school were included as eligible participants. Sampled children were given a study information sheet and a written consent form for them to pass over to one of their parents or primary caregivers. If children were age 10 or older and mentally and intellectually suitable for the survey, they would be asked to respond to the survey. Otherwise, the primary respondent was the parent or the caregiver most familiar with the child’s experiences. Child or parent/caregiver participants were excluded from the study if they were unable to provide consent, or unable to communicate in written or spoken Chinese. All procedures were approved by the Institutional Review Board of the University of Hong Kong/Hospital Authority Hong Kong West Cluster [UW 11-495].

### 2.2. Measures

Child HRQoL was assessed by the 23-item Generic Core Scale of the Chinese version of the Pediatric Quality of Life Inventory (PedsQL) [21], which had four subscales of health-related difficulties: physical functioning, emotional functioning, social functioning, and school functioning. All items were rated on a 5-point scale (from 0 = “never a problem” to 4 = “almost always a problem”). According to the scoring instructions of the PedsQL [21], all item scores were transformed in this study, so that 0 = “100”, 1 = “75”, 2 = “50”, 3 = “25”, and 4 = “0”. Item score were averaged to provide relevant subscale scores, and the mean of all item score was used as the overall HRQoL score. Higher scores reflect fewer difficulties in functioning and thus better HRQoL. The PedsQL has been validated in a Chinese sample and good reliability and validity were demonstrated [21].

Three items were employed to capture children’s ability to communicate with their peers and teachers at school. Participants were asked to rate how well they (or their child) could communicate with their peers and with their teachers (each item was rated on a scale from 1 = “very bad” to 4 = “very well”), and whether they or their child loved to go to school (from 1 = “strongly disagree” to 5 = “strongly agree”). The item scores were summed up to a total ranging from 3 to 13, with higher scores indicating better communication with peers and teachers at school and a greater affinity towards attending school.

The Chinese version of the 12-item Multidimensional Scale of Perceived Social Support (MSPSS-C) was used to assess the level of perceived support from family, friends, and significant others [22]. The items were rated on a 7-point scale. The total score ranged from 12 to 72, with higher scores indicating higher levels of perceived support. The reliability, construct validity, and concurrent validity have been established in the Chinese population in a previous study [22].

The demographic variables of children’s gender, age, their number of siblings, parents’ citizenship in Hong Kong, and parents’ marital status and current employment status were also recorded. Residential instability was indexed with two dimensions adopted from a previous U.S. study [23]: whether or not children were currently living with their parents and the number of times they had moved homes in the year preceding the survey. The average commute time needed for the children to travel between home and school (in minutes), which has been shown to be highly relevant to children’s well-being [16], was also recorded.

### 2.3. Statistical Analysis

The demographic profile of children in this sample and descriptive statistics of the measures used were computed. Between-group differences in demographics were tested by chi-square tests and t-tests. First, a series of multivariable regression analyses were conducted to explore the variables predicting children’s HRQoL. In each of the regression analyses, one independent variable was inputted in one block, while all other variables were inputted in another block so as to control for the effect of other variables. Second, the overall hypothesized model with risk and resilience pathways was explored using path analysis, and the model based on maximum likelihood estimation parameters set in the Mplus program was examined. This was to ensure that estimates would not be biased under conditions of non-normality with potential bias corrected [24]. All hypothesized mediating effects were evaluated with the Sobel test as recommended by previous researchers [25]. Model fit was assessed with three fit indices, including the comparative fit index (CFI), the root mean square error of approximation (RMSEA) and the standardized root mean square of residual (SRMR). Following past research guidelines [26], the model would be considered adequate when CFI > 0.95, RMSEA < 0.06, and SRMR < 0.08.

The path analysis that was conducted using the Mplus version 5.2 (Muthén & Muthén, Los Angeles, CA, USA) [27], and SPSS version 23 (IBM, Armonk, NY, USA) [28] was used for all other statistical analyses. Multi-collinearity was checked before regression analysis and path analysis were conducted. All tests were two-tailed, and the statistical significance level was set at 0.05.

## 3. Results

The final sample consisted of 4807 reports about the participating children (age range = 3–19 years, mean age = 7.47, SD = 2.86, SD: standard deviation), with 809 self-reports from children aged 10 years old or above and 3998 proxy reports from parents or primary caregivers. The response rate was 81.1%.

Table 1 shows the demographic characteristics of the sample. The sample comprised 17.0% 1.75-generation children, 11.0% second-generation children, and 72.0% third-or-higher-generation children. The three groups differed significantly in mean age, number of siblings, parent unemployment, and residential instability dimensions, as well as in the average commute time needed to travel between home and school. There were more cases of single child among the 1.75- and the second-generation than the third-or-higher generation (59.9%, 58.3%, and 44.6% respectively). The 1.75-generation was least likely to live with their parents (94.4%) and needed the longest amount of commute time to travel between their home and school (mean = 59.08 min, SD = 43.30).

Table 2 presents the Cronbach’s alphas, mean scores, and standard deviations of all measures used in this study. Overall, the mean scores of the PedsQL, the MSPSS, and the three items concerning good communication were 77.40 (SD = 15.18), 64.69 (SD = 13.5), and 10.23 (SD = 1.73) respectively. Significant between-group difference was observed in HRQoL related to physical functioning. Third-or-higher generation children demonstrated better physical HRQoL than their 1.75-generation or second-generation counterparts (*p* < 0.001). However, there was no statistically significant between-group difference in the overall HRQoL or in other subscales of HRQoL (*p* > 0.05).

Results from the multivariate regression analysis exploring variables that predict HRQoL are summarized in Table 3. Before adjustment of other variables, lower generations (i.e., 1.75- and second-generations) were predictive to poorer HRQoL when compared to third-or-higher generations. However, the effect became statistically non-significant after controlling for other variables. Older age (β = 0.09, *p* < 0.001), greater number of siblings (β = 0.08, *p* < 0.001), better communication with peers at school (β = 0.30, *p* < 0.001), and higher level of perceived social support (β = 0.08, *p* < 0.001) predicted better HRQoL in our sample controlling for all other variables in the model. On the other hand, having unemployed parent(s) and a longer commute time needed to travel between home and school predicted poorer HRQoL. The whole regression model accounted for 14% of the variability of the PedsQL mean score (*F*-changed = 58.67, *p* < 0.001).

**Figure 1:**
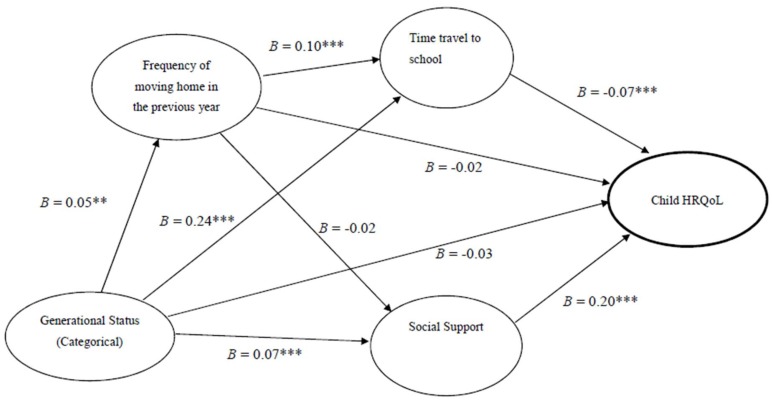
The Path Model Explaining Child Health-Related Quality of Life (HRQoL) with Generational Status, Residential Instability, and Social Support. Note: ** *p* < 0.01; *** *p* < 0.001.

Findings from the path analysis are shown in Figure 1 and Table 4. The three model fit indices (CFI = 0.99; RMSEA = 0.01; SRMR = 0.01) indicated that the model was adequately fit. The χ^2^ of model fit was 544.7 (degree of freedom=10, *p* < 0.001). Residuals of the final model were plotted and checked, and normal distribution was demonstrated. As shown in Table 4, both commute time needed to travel to school (β = −0.07, *p*< 0.001) and perceived social support (β = 0.20, *p* < 0.001) had significant direct effects on HRQoL, but not generational status or frequency of moving homes in the previous year. However, the effects of generational status on HRQoL was significantly mediated by time needed to commute between home and school and perceived social support (β = −0.03, *p* < 0.001). In other words, the effects of being in the 1.75-generation or the second-generation groups on HRQoL were reduced when commute time between home and school, frequency of moving homes, and social support were included in the model. Specifically, the indirect effect of frequency of moving homes on HRQoL was via increased commute time (β = 0.10, *p* < 0.001).

## 4. Discussion

In a large stratified sample of school-aged children in Hong Kong, this study aimed to integrate and move forward previous literature on risk factors associated with immigration contexts and resilience dimensions by examining the effects of generational status on children’s HRQoL in three distinct groups of immigrant children, and the mediating roles of residential instability and social support. As demonstrated by the statistical findings, significantly lower HRQoL related to physical functioning was observed in the 1.75-generation and the second-generation groups when compared to the third-or-higher generation. The association between generational status and HRQoL was mediated by commute time needed to travel between home and school, the frequency of moving home via increased commute time, and perceived social support.

Echoing previous studies on immigrants [2,9], the present findings showed that the third-or-higher generation had better well-being in terms of physical HRQoL than the other two groups. This finding is in line with the literature on migration and generational effects [1,2,3,9]. Furthermore, results demonstrated that the association between generational status and the HRQoL can be partially explained by risk factors relevant to residential instability that typically characterizes cross-boundary immigrant families, namely the time needed to commute between home and school, as well as the number of times their families moved homes in the past year.

Indeed, the longest commute time needed to travel between home and school was observed among the 1.75-generation, whose HRQoL was significantly poorer than the third-or-higher generation. This group of children born to two non-local parents might live in cities near the border between Hong Kong and mainland China (e.g., Shenzhen), and thus need to travel cross-border every school day [15]. These long journeys to school, especially those on uncomfortable buses, often have a negative impact on children’s physical health and academic achievement by increasing the children’s stress levels and reducing time for extra-curricular activities [16].

The poorer HRQoL related to physical functioning among the 1.75-generation might also be attributed to chronic stress stemming from the greater number of times their families moved homes. Frequent mobility has not only been associated with physical and mental health problems and disruptions in access to healthcare services [29], but in this study it was related to commute burden such that it forecasted an increase in commute time needed to travel between home and school. Commute burden may give rise to heightened fatigue and less time for children to engage in activities with peers to foster healthy social development, and thus may presage a trajectory of poorer overall HRQoL.

Interestingly, despite the poorer physical HRQoL reported by the 1.75-generation and the second-generation groups compared to their third-or-higher generation counterparts, the former two generation groups reported higher than expected levels of social support from families, friends, and significant others. Furthermore, our results indicated that the link between generational status and HRQoL was partially explained by social support as a pathway independent from risk factors relevant to the physical context of immigrant families. Disadvantaged immigrant groups do tend to lack adequate social resources [20], but there is individual variability in the acculturation experiences of immigrant families and some may be able to acquire effective social support during their integration process [30]. The observed pattern in our sample may reflect a relatively successful adaption to mainstream culture and the presence of other processes that promote social integration [30], as well as a welcoming environment provided by the government and the community into which they immigrate [31]. Specifically, since most of the 1.75- and the second-generation children were studying in schools located in districts close to the border between mainland China and Hong Kong to reduce commute time, their schoolteachers may have a better understanding of the acculturation difficulties these children experienced, and may have provided extra support to help them adjust to the school system in Hong Kong.

### Limitations

Several limitations existed in this study. Potential biases are inherent in self-reports and proxy reports with regards to the children’s experiences, despite our attempts to ask parents or caregivers most familiar with the children to be respondents. The missing rate of the item about gender was also surprisingly high (11.9%). Although the reluctance to report gender undoubtedly needs further investigation, we believe one reason behind could be the feelings of insecurity among participants to report such information. Future studies should emphasize that no personal information would be investigated individually, so as to provide a more secure environment for participants. Multiple imputation technique might also be of help to treat the missing data in future research. Finally, the conceptualization of residential instability was adopted from a previous study and did not include more dimensions of the concept. Future studies could include more aspects of the concept so as to help extend our knowledge in its influence on children.

## 5. Implications

To our knowledge, this is the first study to examine the links between generational status and HRQoL among children in the unique context of cross-boundary families in Hong Kong, as well as plausible risk and resilience mechanisms by which generational status can have implications for children’s well-being and quality of life. These findings helped identify plausible risk factors that require intervening to mitigate the burden of first- and second-generation contexts on immigrant children’s development (e.g., the burden of medical service utilization among lower generations as demonstrated by the literature [12,13]), and also provide viable social capital target dimensions to promote adaptive assimilation. Furthermore, the unique stressors experienced by first- and second-generation groups while families navigate citizenship hurdles and permanent residency offer insight to help precisely optimize interventions for each group. When social resources are at equal levels across distinct generation groups, the disadvantaged groups appear to be reach better HRQoL than their higher generation counterparts. Our findings provide key insights into the possible suppression of the generational effects on children’s HRQoL by likely modifiable factors, and these findings might lend utility to help address challenges experienced by international immigrant developmental populations, such as the 5.5 million children of undocumented immigrant parents in the United States [15]. These immigrant children are challenged with health problems but unfortunately have been a blind spot for policymakers and researchers [32]. They are not protected with affordable health care as other local children are [33]. It has been a global public health challenge that future policy making and research should integrate rigorous investigations of physical, familial, and psychosocial risk factors as well as resilience dimensions can help foster healthy development amongst culturally assimilating children.

## 6. Conclusions

To our knowledge, this is the first study to examine the links between generational status and HRQoL among children in the unique context of cross-boundary families and anchor babies in Hong Kong, as well as plausible risk and resilience processes to help explain how immigration context has implications for children’s well-being. Our findings shed lights on the generational effects on children’s well-being by likely modifiable level of social support and residential stability. Future research that integrates rigorous investigations of physical, familial, and psychosocial risk factors as well as resilience dimensions can help foster healthy development amongst culturally assimilating children.

## Figures and Tables

**Table 1:** Demographic characteristics of the child sample.

Characteristic	Frequency (Percentage %)				*p*-Value
	All (*N* = 4807)	By Generational Status			
		1.75-Generation (*n* = 818)	Second-Generation (*n* = 531)	Third-or-Higher-Generation (*n* = 3458)	
Gender					0.14
Male	2130 (44.1)	302 (34.7)	218 (40.1)	1610 (44.4)	
Female	2009 (44.0)	321 (40.4)	210 (39.2)	1478 (44.2)	
Missing	668 (11.9)	195 (24.9)	103 (20.8)	370 (11.4)	
Age [mean (SD)]	7.47 (2.86)	6.59 (2.35)	6.69 (2.67)	7.50 (2.87)	0.03
Age range	3–19	3–15	3–16	3–19	
No. of siblings					<0.001
None	2432 (45.2)	488 (59.9)	297 (58.3)	1647 (44.6)	
One	1902 (42.6)	245 (30.1)	198 (34.2)	1459 (43.0)	
Two or more	473 (12.2)	85 (10.0)	36 (7.5)	352 (12.4)	
Unemployed parent(s)	387 (8.1)	75 (9.2)	63 (11.9)	249 (7.2)	0.001
Living with parents	4643 (97.2)	768 (94.4)	517 (97.8)	3358 (97.2)	0.002
Frequency of moving home in the previous year [mean (SD)]	0.16 (0.48)	0.21 (0.48)	0.17 (0.45)	0.16 (0.48)	0.002
Average commute time needed to travel between home and school (one-way) (min) [mean (SD)]	34.32 (28.89)	59.08 (43.30)	37.33 (33.26)	28.01 (27.93)	<0.001

**Table 2:** Mean scores and standard deviations of the measures among the child sample.

Measure	Number of Items	Cronbach’s Alpha	Mean (SD)				*p-*Value
			All (*N* = 4807)	By Generational Status			
				1.75-Generation (*n* = 818)	Second-Generation (*n* = 531)	Third-or-Higher-Generation (*n* = 3458)	
Child health-related quality of life (HRQoL)	23	0.92	77.40 (15.18)	74.26 (16.88)	75.15 (16.51)	77.51 (15.10)	0.063
Physical functioning	8	0.87	82.83 (18.48)	77.34 (21.47)	78.91 (22.09)	83.02 (18.32)	<0.001
Emotional functioning	5	0.83	76.09 (18.61)	75.84 (17.53)	75.22 (18.06)	76.11 (18.64)	0.307
Social functioning	5	0.80	78.49 (20.43)	74.83 (21.40)	76.51 (20.06)	78.61 (20.41)	0.276
School functioning	5	0.78	68.87 (19.76)	67.08 (19.95)	66.79 (19.88)	68.95 (19.75)	0.694
Good communication with peers and teachers	3	0.68	10.23 (1.73)	10.29 (1.70)	10.28 (1.61)	10.22 (1.73)	0.072
Social support	12	0.95	64.69 (13.58)	66.37 (12.27)	64.56 (12.98)	64.65 (13.61)	<0.001
Family	4	0.93	22.70 (4.74)	23.32 (4.31)	22.46 (4.57)	22.69 (4.76)	0.01
Friends	4	0.94	20.83 (5.28)	21.27 (4.66)	20.73 (4.94)	20.82 (5.30)	<0.001
Significant other	4	0.91	21.17 (5.18)	21.65 (4.67)	21.43 (4.75)	21.16 (5.20)	<0.001

Note: Child health-related quality of life was measured by Pediatric Quality of Life Inventory (PedsQL); and social support was measured by Multidimensional Scale of Perceived Social Support (MSPSS). *p*-Value refers to the F-statistics in ANOVA.

**Table 3:** Standardized Coefficients of the Regression Analysis for Variables Predicting the Overall Score of the Pediatric Quality of Life Inventory (PedsQL) (*N* = 4807).

Variable	B (Crude)	*p*-Value	β [95% Confidence Interval]	β (Adjusted)	*p*-Value
Generational status ^a^	−0.03 *	0.02	−0.28 [−1.46, 0.898]	−0.01	0.64
Gender ^b^	0.03	0.08	−0.19 [−1.07, 0.87]	−0.003	0.84
Age (year)	0.05 **	0.003	0.46 [0.29, 0.63]	0.09	<0.001
No. of siblings	0.08 ***	<0.001	1.81 [1.08, 2.54]	0.08	<0.001
Unemployed parent(s) ^c^	−0.07 ***	<0.001	−2.85 [−4.67, −1.03]	−0.05	0.002
No. of times moved home in the previous year	−0.03 *	0.03	−0.63 [−1.74, 0.47]	−0.02	0.26
Time needed to travel from home to school (one-way, in minute)	−0.08 ***	<0.001	−0.03 [−0.04, −0.01]	−0.05	0.001
Good communication	0.32 ***	<0.001	2.74 [2/42, 3.05]	0.30	<0.001
Social support	0.19 ***	<0.001	0.10 [0.05, 0.14]	0.08	<0.001
R^2^			0.135	0.135	
*F* for change in R^2^			58.673	58.673	
*p*-Value			<0.001	<0.001	

Note. * *p* < 0.05. ** *p* < 0.01. *** *p* < 0.001. ^a^ Generational status: referent group = third-or-higher-generation; comparison group = 1.75-generation + second-generation. ^b^ Gender: referent group = female; comparison group = male. ^c^ Unemployed parents: referent group = employed parents; comparison group = unemployed parents.

**Table 4:** Standardized coefficients of the path model (*N* = 4807).

Outcome Variable	Exogenous Variables	Effects		
		Direct	Indirect	Total
Child HRQoL				
	Social Support	0.20 ***	–	–
	Time travel to school	−0.07 ***	–	–
	Frequency of moving home in previous year	−0.02	–	–
	Generational status ^a^	−0.03	–	–
Time travel to school				
	Generational status ^a^	0.24 ***	–	–
	Frequency of moving home in previous year	0.10 ***	–	–
Social Support				
	Generational status ^a^	0.07 ***	–	–
	Frequency of moving home in previous year	−0.02	–	–
Generational status				
	Frequency of moving home in previous year	0.05 **	–	–
Child HRQoL				
	Generational status ^a^	−0.03	–	−0.03 ***
	Via Social Support	−	0.01 ***	–
	Via Time travel to school	−	−0.02 ***	–

Note. ** *p* < 0.01. *** *p* < 0.001. Child HRQoL = Child health-related quality of life. ^a^ Generational status: referent group (1) = third-or-higher-generation; comparison group (2) = 1.75-generation + second-generation.

## References

[1] Garcia C.C., Lamberty G., Jenkins R., McAdoo H.P., Cmic K., Wasik B.H., Vazquez G.H. (1996). An integrative model for the study of developmental competencies in minority children. Child Dev..

[2] Finch B.K., Hummer R.A., Kol B., Vega W.A. (2001). The role of discrimination acculturative stress in the physical health of Mexican-origin adults. Hisp. J. Behav. Sci..

[3] Harker K. (2000). Immigrant generation, assimilation, and adolescent psychological well-being. Soc. Forces.

[4] Kaufman P., Chavez L., Lauen D., Carroll C.D. (1988). Generational Status and Educational Outcomes among Asian and Hispanic 1988 Eighth Graders. http://nces.ed.gov/pubs99/1999020.pdf.

[5] Durazo E.M., Wallace S.P. (2014). Access to health care across generational status for Mexican-origin immigrants in California. FACTS Rep..

[6] Hummer R.A., Powers D.A., Pullum S.G., Gossman G.L., Frisbie W.P. (2007). Paradox found (again): Infant mortality among the Mexican-origin population in the United States. Demography.

[7] Alegria M., Mulvaney-Day N., Torres M., Polo A., Cao Z., Canino G. (2007). Prevalence of psychiatric disorders across Latino subgroups in the United States. Am. J. Public Health.

[8] Mehta S. Health Needs Assessment of Asian People Living in the Auckland Region. http://www.countiesmanukau.health.nz/assets/About-CMH/Performance-and-planning/health-status/2012-health-needs-of-asian-people.pdf.

[9] Wang C.C., Castaneda-Sound C. (2008). The role of generational status, self-esteem, academic self-efficacy, and perceived social support in college students’ psychological well-being. J. Coll. Couns..

[10] Rumbaut R.G. (2004). Ages, life stages, and generational cohorts: Decomposing the immigrant first and second generations in the United States. Int. Migr. Rev..

[11] Lederer J. (2013). “Anchor baby”: A conceptual explanation for pejoration. J. Pragmat..

[12] BeLue R., Miranda P.Y., Elewonibi B.R., Hillemeier M.M. (2014). The Association of Generation Status and Health Insurance Among US Children. Pediatrics.

[13] Huang Z.J., Yu S.M., Ledsky R. (2006). Health status and health service access and use among children in the U.S. immigrant families. Am. J. Public Health.

[14] Suarez-Orozco C., Yoshikawa H. (2013). Undocumented status: Implications for child development, policy, and ethical research. New Dir. Child Adolesc. Dev..

[15] Yuen C.Y.M. (2011). Towards inclusion of cross-boundary students from Mainland China in educational policies and practices in Hong Kong. Educ. Citizensh. Soc. Justice.

[16] Scottish Executive Central Research Unit (2016). Review of Research on School Travel. https://www2.gov.scot/Publications/2002/05/14690/4177.

[17] Oppedal B., Roysamb E., Sam D.L. (2004). The effect of acculturation and social support on chance in mental health among young immigrants. Int. J. Behav. Dev..

[18] Salinero-Fort M.A., Otero-Sanz L., Martin-Madrazo C., Burgos-Lunar C., Chico-Moraleja B.M., Rodes-Soldevila B., Jimenez-Garcia R., Gomez-Campelo P. (2011). The relationship between social support and self-reported health status in immigrants: An adjusted analysis in the Madrid Cross Sectional Study. BMC Fam. Pract..

[19] Wong W.K.F., Chou K.L., Chow N.W.S. (2012). Correlates of quality of life in new migrants to Hong Kong from mainland China. Soc. Indic. Res..

[20] Herz M., Johansson T. (2012). The experience of being stopped: Young immigrants, social exclusion and strategies. Young.

[21] Lau J.T., Yu X.N., Chu Y., Shing M.M., Wong E.M., Leung T.F., Li C.K., Fok C.K., Mak W.W. (2010). Validation of the Chinese version of the Pediatric Quality of Life Inventory (PedsQL) cancer module. J. Pediatr. Psychol..

[22] Chou K.L. (2000). Assessing Chinese adolescents’ social support: The Multidimensional Scale of Perceived Social Support. Pers. Individ. Differ..

[23] Turner H.A., Finkelhor D., Hamby S.L., Shattuck A. (2013). Family structure, victimization, and child mental health in a nationally representative sample. Soc. Sci. Med..

[24] Efron B., Tibshirani R.J. (1993). An Introduction to the Bootstrap.

[25] MacKinnon D.P., Lockwood C.M., Hoffman J.M., West S.G., Sheets V. (2002). A comparison of methods to test mediation and other intervening variable effects. Psychol. Methods.

[26] Hu L.T., Bentler P.M. (1999). Cutoff criteria for fit indices in covariance structure analysis: Conventional criteria versus new alternatives. Struct. Equ. Model..

[27] Methen L.K., Muthen B.O. (2007). Mplus User’s Guide.

[28] George D., Mallery P. (2016). IBM SPSS Statistics 23 Step by Step: A Simple Guide and Reference.

[29] Kingsley G.T., Jordan A., Traynor W. (2012). Addressing residential instability: Options for cities and community initiatives. Cityscape.

[30] Berry J.W. (1997). Immigration, acculturation, and adaptation. Appl. Psychol..

[31] Haller W., Landolt P. (2005). The transnational dimensions of identity formation: Adult children of immigrants in Miami. Ethn. Racial Stud..

[32] Miller-Thayer J. (2010). Health Migration: Crossing Borders for Affordable Health Care. Field Actions Sci. Rep..

[33] Wickramage K., Vearey J., Zwi A.B., Robinson C., Knipper M. (2018). Migration and health: A global public health research priority. BMC Public Health.

